# Serum Leptin Levels in Polycystic Ovary Syndrome and Its Relationship with Metabolic and Hormonal Profile in Pakistani Females

**DOI:** 10.1155/2014/132908

**Published:** 2014-12-23

**Authors:** Mukhtiar Baig, Rehana Rehman, Saba Tariq, Syeda Sadia Fatima

**Affiliations:** ^1^Department of Clinical Biochemistry, Faculty of Medicine, Rabigh, King Abdulaziz University, Jeddah 21589, Saudi Arabia; ^2^Department of Biological and Biomedical Sciences, Aga Khan University, Stadium Road, Karachi 74800, Pakistan; ^3^Department of Pharmacology, University Medical & Dental College, Faisalabad 38000, Pakistan

## Abstract

The study aimed to investigate the levels of serum leptin in PCOS females and to correlate it with metabolic and hormonal parameters. Sixty-two PCOS and ninety normal cycling (NC) females with matched age and body mass index (BMI) were recruited for this cross-sectional study. Serum leptin, FSH, LH, E2, free testosterone, progesterone, thyroid profile, and FBG levels were measured. The mean leptin levels in PCOS and NC were not significantly different (45.56 ng/mL ± 1.49 vs 41.78 ± 1.31 ng/mL, *P* > 0.05); however, leptin levels showed a strong correlation with BMI in PCOS and NC group (*r* = 0.77, *P* < 0.0001; *r* = 0.82, *P* < 0.0001, resp.). High E2 levels in NC had a significant correlation with leptin whereas FBG correlated with leptin in PCOS (*r* = 0.51, *P* = 0.005). TSH had a substantial correlation (*r* = 0.49, *P* < 0.005; *r* = 0.69, *P* < 0.005) in PCOS and NC, respectively. There was no significant difference found in circulating leptin concentration between PCOS and NC subjects. Leptin levels in PCOS were related with metabolic impairments manifested by disturbance in FBG levels and impairment of reproductive functions in terms of reduced E2 secretion.

## 1. Introduction

Polycystic ovaries have been reported in 20–30% of women and possibly 70–80% of these polycystic ovaries demonstrate some common features like menstrual irregularity, subfertility, obesity, hirsutism, acne, and abnormal biochemistry with raised serum testosterone, androstenedione, insulin, and luteinizing hormone level [1]. It is frequently seen that asymptomatic females complain of unrelated problem like weight gain, and PCOS is sometimes inadvertently diagnosed by ultrasonic examination in such females.

Leptin, a product of OB gene, is produced in adipose tissues and has a long list of endocrine functions besides being responsible for causing obesity [2]. The relationship of leptin and obesity is well known [2]. Additionally, higher and lower levels of leptin are also related with infertility but the mechanism of involvement is still undiscovered [3, 4]. Its role in reproductive physiology and the pathogenesis of PCOS has been studied in bioactive leptin deficient rats [5]. It was observed that treatment with recombinant leptin helped them to resume fertility [6–8] but the underlying mechanisms are still not clear.

The etiology of PCOS is not known. The presence of obesity, insulin resistance, hyperandrogenemia, and infertility in PCOS resemble those found in “ob/ob” leptin deficient rats [7]. This association motivated us to design this study to investigate the concentration of serum leptin in PCOS females and to correlate it with metabolic and hormonal parameters in Pakistani females.

## 2. Subjects and Methods

Present cross-sectional study was conducted in the Department of Biochemistry, University of Karachi, Karachi, Pakistan, and PCOS subjects were selected from the Out Patient Department of Gynecology and Obstetrics as well as Infertility Clinic of Jinnah Postgraduate Medical Centre (JPMC), Karachi, Pakistan. Sixty-two PCOS subjects (mean age 29.94 ± 2.51 years) who completely fulfilled our selection criteria and ninety (90) fertile, healthy female subjects (mean age 28.87 ± 3.64 years), having regular menstrual cycle without clinical evidence of hyperandrogenism and ultrasonographic finding of PCOS, were selected from the general population.

### 2.1. Criteria for Selection of PCOS Patients

The diagnostic criteria of Rotterdam consensus group [9] were used to select PCOS subjects. Females with lacking continuity or regularity in menses or oligomenorrhea (menses failure for 35–182 days) or amenorrhea (menses failure for >182 days), hirsutism and laboratory confirmation of excess of androgen (i.e., high serum free testosterone level), ultrasonographic records confirming not less than 12 follicles (two to nine mm in diameter) placed peripherally around a dense core of ovarian stroma or spread throughout, and enhanced quantity of stroma were registered in the study.

### 2.2. Exclusion Criteria

Subjects in both groups having systemic illness, including thyroid disorder, Cushing syndrome, congenital adrenal hyperplasia, systemic inflammatory diseases, acromegaly, hyperprolactinaemia, functional hypothalamic amenorrhea, diabetes mellitus, using oral contraceptive pills, or any other hormonal medication, were excluded from the study.

In NC females blood samples were collected in follicular phase of menstrual cycle, while in PCOS patients samples were collected independently of day of menses because of irregularity in menstrual cycle.

All patients and NC women were provided with informed consent for the study and questionnaires regarding their demographic data and signs and symptoms were filled. Height and weight of all participants were evaluated and BMI was calculated [BMI = body weight (kg)/height (m^2^)]. The Board of Advanced Studies and Research (BASR), University of Karachi, had given approval to conduct this study according to Helsinki declaration of human rights. All participants of the study had also given their written informed consent.

### 2.3. Laboratory Measurement

Blood samples were obtained between 08:00 AM and 10:00 AM after 12 hours of fasting. Serum was separated and stored at −20°C until further analyses. Follicle stimulating hormone (FSH), luteinizing hormone (LH), free testosterone, estradiol (E2), progesterone, free thyroxin (FT4), free triiodothyronine (FT3), and thyroid stimulating hormone (TSH) levels were measured with standard radioimmunoassay (RIA) on gamma counter (Capintec, USA). Immunoradiometric (IRMA) kit (Diagnostic Systems Laboratories, USA) was used for assaying serum leptin levels. The sensitivity of leptin IRMA kit (lowest detectable limit) was 0.1 ng per milliliter and intra- and interassay coefficients of variations were less than 8%. Fasting blood glucose (FBG) concentrations were determined by glucose oxidase method.

### 2.4. Statistical Analysis

Statistical analysis was executed by SPSS 16 (SPSS inc., Chicago, IL) program. Student's *t*-test was employed for calculating comparison between groups and *P* < 0.05 was taken as significant. Pearson correlation analysis was used to determine linear correlations between the variables.

Analysis of covariance (ANCOVA) was applied to find out the correlation of serum leptin levels with other variables (FBG, TSH, E2, and testosterone) after adjusting BMI (because leptin levels and BMI are positively related with each other).

## 3. Results

The comparison of mean age, BMI, leptin, and hormonal and metabolic parameters (FSH, LH, free testosterone, prolactin, T3, T4, TSH levels, and FBG) of PCOS and NC is shown in Table 1. The mean leptin levels were 45.56 ± 1.49 and 41.78 ± 1.31 ng/mL in patients with PCOS and NC, respectively (*P* > 0.05). In PCOS patients, mean FBG level was considerably elevated compared to controlled group [*P* < 0.001] (Table 1). Serum FSH, LH, and E2 concentrations were considerably lower in patients with PCOS (*P* < 0.001). Serum LH/FSH ratio, testosterone, and progesterone concentration were considerably elevated in the PCOS subjects as compared to NC women (*P* < 0.001). There was no noteworthy variation in prolactin, T3, T4, and TSH between the groups (Table 1).

**Figure 1:**
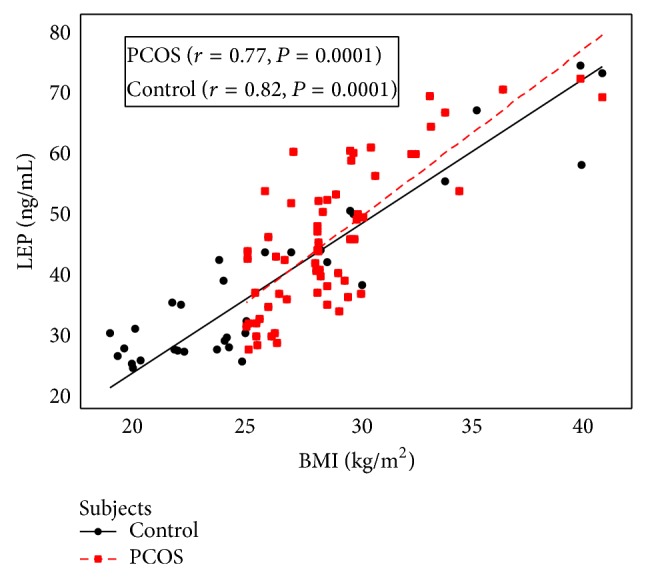
Correlation between serum leptin concentration and BMI in PCOS and control women.

**Figure 2:**
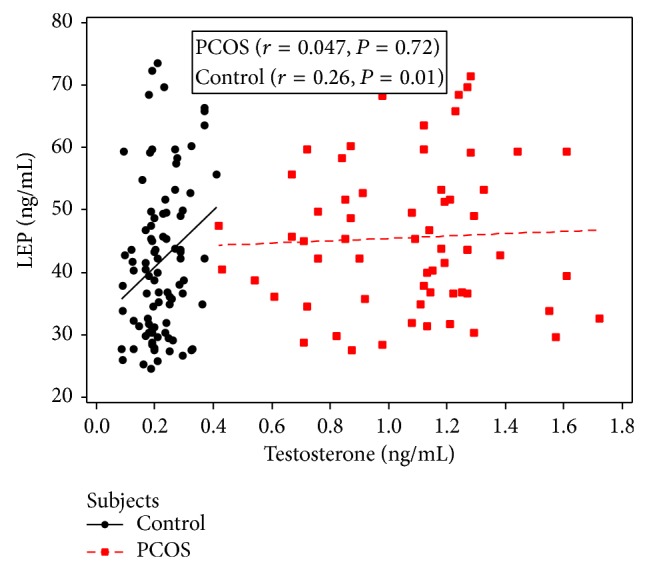
Correlation between serum leptin concentration and testosterone in PCOS and age and BMI matched control women.

A positive correlation of serum leptin levels with FBG was observed (*r* = 0.51, *P* < 0.005). Serum leptin was associated with TSH and E2 in NC females (*r* = 0.69, *P* < 0.005; *r* = 0.79, *P* < 0.0001, resp.), while it was linked with only TSH in PCOS patients (*r* = 0.49, *P* < 0.005) (Table 2). A substantial positive correlation was found between serum leptin levels and BMI in the PCOS subjects and controlled group (*r* = 0.77, *P* < 0.0001 and *r* = 0.82, *P* < 0.0001 resp.) (Figure 1). The testosterone level was notably correlated with leptin in NC group (*r* = 0.26, *P* = 0.01), while no link was found in PCOS patients (*r* = 0.047, *P* = 0.72) (Figure 2).

Nevertheless, when the correlation of serum leptin concentration with other variables (FBG, TSH, estradiol, and testosterone) was recalculated with ANCOVA after adjusting BMI, the significant correlation between leptin and FBG became nonsignificant and other correlations remained significant.

## 4. Discussion

Insulin resistance, obesity, and distorted endocrine particularly ovarian functions are likely to take place in PCOS [10, 11]. These features manifest with advancement of age and gradual accumulation of adiposity [12, 13]. Leptin seems to be directly related to obesity by maintaining energy homeostasis with decreased food intake and increased energy expenditure [14].

Our study observed higher serum leptin levels in PCOS patients compared to NC females but it was not significantly different. These results are comparable with Telli et al. [7]; they also reported nonsignificant higher leptin levels in PCOS patients as compared to NC. Few other studies have also reported no significant difference in serum leptin levels of PCOS subjects with age and BMI matched controls [15, 16]. Olszanecka-Glinianowicz et al. (2013) documented considerably higher serum leptin level in PCOS subjects compared to age and BMI matched NC group. Furthermore, they observed that the serum leptin level was significantly higher in obese PCOS patients compared to lean PCOS and obese non-PCOS patients [17]. Few other studies also described significantly elevated levels of leptin in PCOS [11, 18].

In present study, serum leptin level is considerably correlated with BMI in both groups and this result is corroborated with other studies [2, 11, 19]. The correlation of serum leptin with the quantity of adipose tissue in PCOS has also been supported by Kazmi et al. (2013) [20].

Fertility in females is regulated by hypothalamo-pituitary-ovarian feedback axis which is under control of gonadotropin releasing hormone (GnRH). Under its influence, anterior pituitary gonadotrophs secrete LH and FSH in a pulsatile fashion which stimulates ovaries to produce sex hormones. Leptin acts as a metabolic controller of reproductive capacity by giving information about the quantity of energy stored in the body to GnRH neurons [21]. High leptin levels also contribute in the pathophysiology of PCOS by increasing Gn RH release, stimulating pituitary gonadotrophs or the ovaries [22].

The high LH/FSH ratio of PCOS found in our study is comparable with other [7] whereas low LH/FSH ratio was observed by other [2]. We observed no correlation of leptin with FSH or LH levels and LH/FSH ratio in PCOS and it supports the hypothesis that role of leptin in pituitary ovarian dysfunction for reproductive disorder in PCOS patients should be explored further as has been proposed by others [7].

The ovarian and endometrial cycle is bridged by production of E2 from granulosa cells which cause proliferation of epithelial and stromal components of endometrium [23]. Leptin plays an imperative role in endocrine functions of ovaries by its role in gametogenesis as well as steroidogenesis [2]. High E2 levels in NC and its strong correlation with leptin emphasize the role of leptin in reproductive endocrine functions of normal women. Inadequate levels of E2 suggestive of disturbance in the endocrine milieu in PCOS were not associated with leptin levels. Presence of leptin in NC women enhanced ovarian steroidogenesis represented by increased E2 in this group whereas neuroendocrine disturbance in the form of a low FSH, LH, and high LH-FSH ratio was observed in PCOS.

Alteration of androgen production rate in PCOS is the result of adrenal and ovarian secretion and conversion from precursors in adipose tissue and skin. The extent of androgen concentration imitates the degree of sympathoexcitation and testosterone production which is associated with the degree of PCOS severity, phenotypic abnormalities, and defective OB production [24]. The correlation of leptin with testosterone in NC females suggests the role of leptin in androgen production which is supported by others [25]. The relative high testosterone level in PCOS was not associated with leptin as has been observed by others [4]. Low FSH, LH, and E2 and relatively high testosterone levels in PCOS indicate resistance at receptor level of pituitary, adrenals, and gonads which needs to be explored at molecular levels.

The current study observed that the serum levels of FSH, LH, and E2 were significantly low, while LH/FSH ratio, testosterone, progesterone, and FBG levels were considerably high in PCOS subjects compared to NC females. The reason for significant difference in hormonal levels in both groups could be due to difference in blood sample collection. The blood samples collected from NC subjects on 12th day of the menses and at this time FSH and LH levels are at their peak and E2 level is also increased while blood samples in PCOS subjects were collected irrespective of their menstrual cycle. In present study lack of correlation with FSH, LH, LH/FSH ratio, and E2 does not support the role of leptin on gonadotropin secretion and ovarian steroidogenesis in PCOS.

Leptin is considered to play a role in conservation of energy and maintenance of body weight by neuroendocrine mechanisms. The physiological mechanisms for thyroid hormone-induced alteration in serum leptin are not well known. It has been observed that leptin deficient patients cannot acclimatize in cold environment because of low T3, T4 and afflicted control of hypothalamo-pituitary-thyroid axis [26]. In our study control subjects had high TSH which is strongly correlated with leptin. In another study, serum leptin levels were correlated with BMI and TSH in both hypothyroid and hyperthyroid patients that were independent of thyroid hormones [27].

Leptin resistance impairs peripheral glucose homeostasis by decreasing the hypothalamic response to insulin that may lead to the onset of type 2 diabetes [28]. The cross talk of insulin and leptin can be explained by high levels of fasting blood glucose in PCOS that correlated with leptin. The association of leptin with hyperglycemia and its significant correlation with serum glucose and insulin levels in PCOS thus explain its role in metabolic impairments, which may occur due to insulin resistance. The correlation of TSH with leptin was an accidental finding at the end of the day, which needs to be investigated for further research on leptin and its effects on obesity and metabolic impairments.

The study is limited in terms of its small sample size, and lack of categorization of obese and non-obese subgroups in PCOS and NC females. It also did not measure body fat, serum insulin and androstenedione levels to establish their relationship with the anthropometric, metabolic and hormonal impairments likely to occur with leptin dysregulation. Future longitudinal studies with larger cohorts are required to confirm these findings.

## 5. Conclusion

There was no significant difference found in circulating leptin concentration between PCOS and NC subjects. A strong correlation of leptin with BMI was observed in both groups and leptin levels in PCOS were related with metabolic impairment manifested by disturbance in FBG levels and impairment of reproductive functions in terms of reduced E2 secretion.

## Figures and Tables

**Table 1:** Comparison of study parameters in PCOS and control subjects.

Parameters	PCOS subjects *N* = (62)	Control subjects *N* = (90)
Age (years)	29.94 ± 2.51	28.87 ± 3.14
BMI (Kg/m^2^)	28.83 ± 0.41	27.86 ± 0.48
Leptin (ng/mL)	45.56 ± 1.49	41.78 ± 1.31
Follicle stimulating hormone (IU/L)	2.17 ± 0.052	10.22 ± 0.25^*^
Luteinizing hormone (IU/L)	8.00 ± 0.23	25.50 ± 0.47^*^
LH/FSH ratio	3.35 ± 0.08	2.21 ± 0.13^*^
Testosterone (ng/mL)	1.07 ± 0.03	0.22 ± 0.01^*^
Estradiol (pg/mL)	169.22 ± 4.21	271.43 ± 6.93^*^
Progesterone (ng/mL)	1.99 ± 0.08	0.34 ± 0.02^*^
Prolactin (ng/mL)	10.37 ± 0.22	9.78 ± 0.28
Free T_3_ (ng/dL)	1.06 ± 0.02	1.04 ± 0.01
Free T_4_ (pg/mL)	2.04 ± 0.03	2.10 ± 0.02
Thyroid stimulating hormone (mIU/L)	2.06 ± 0.06	2.19 ± 0.08
Fasting blood glucose (mg/dL)	93.81 ± 1.79	80.12 ± 1.38^*^

Results are shown as mean ± SEM; ^*^ *P* < 0.001.

**Table 2:** Correlation coefficient of serum leptin with different variables in PCOS and control subjects.

Parameters	PCOS subjects *N* = (62)	Control subjects *N* = (90)
Follicle stimulating hormone (IU/L)	−0.001	0.09
Luteinizing hormone (IU/L)	−0.02	0.13
Estradiol (pg/mL)	−0.08	0.79^**^
Progesterone (ng/mL)	−0.07	0.01
Prolactin (ng/mL)	−0.12	0.10
Free T_3_ (ng/dL)	−0.16	0.05
Free T_4_ (pg/mL)	0.23	−0.15
Thyroid stimulating hormone (mIU/L)	0.49^*^	0.69^*^
Fasting blood glucose	0.51^*^	0.24

Values are given as correlation coefficient (*R*); ^*^ *P* < 0.005; ^**^ *P* < 0.0001.

## References

[1] Usmani A., Rehman R., Akhtar Z. (2014). Association of body mass index and dietary habits with ovarian and uterine morphology with subfertile polycystic ovarian syndrome. *Journal of Postgraduate Medical Institute*.

[2] Chakrabarti J. (2013). Serum leptin level in women with polycystic ovary syndrome: correlation with adiposity, insulin, and circulating testosterone. *Annals of Medical and Health Science Research*.

[3] Barash I. A., Cheung C. C., Weigle D. S., Ren H., Kabigting E. B., Kuijper J. L., Clifton D. K., Steiner R. A. (1996). Leptin is a metabolic signal to the reproductive system. *Endocrinology*.

[4] Brzechffa P. R., Jakimiuk A. J., Agarwal S. K., Weitsman S. R., Buyalos R. P., Magoffin D. A. (1996). Serum immunoreactive leptin concentrations in women with polycystic ovary syndrome. *The Journal of Clinical Endocrinology & Metabolism*.

[5] Drel V. R., Mashtalir N., Ilnytska O., Shin J., Li F., Lyzogubov V. V., Obrosova I. G. (2006). The leptin-deficient (*ob/ob*) mouse: a new animal model of peripheral neuropathy of type 2 diabetes and obesity. *Diabetes*.

[6] Brannian J. D., Schmidt S. M., Kreger D. O., Hansen K. A. (2001). Baseline non-fasting serum leptin concentration to body mass index ratio is predictive of IVF outcomes. *Human Reproduction*.

[7] Telli M. H., Yildirim M., Noyan V. (2002). Serum leptin levels in patients with polycystic ovary syndrome. *Fertility and Sterility*.

[8] Blüher S., Mantzoros C. S. (2007). Leptin in reproduction. *Current Opinion in Endocrinology, Diabetes and Obesity*.

[9] Rotterdam ESHRE/ASRM-Sponsored PCOS Consensus Workshop Group (2004). Revised 2003 consensus on diagnostic criteria and long-term health risks related to polycystic ovary syndrome. *Human Reproduction*.

[10] Baranova A., Tran T. P., Afendy A. (2013). Molecular signature of adipose tissue in patients with both non-alcoholic fatty liver disease (NAFLD) and polycystic ovarian syndrome (PCOS). *Journal of Transplant Medicine*.

[11] El-Gharib M. N., Badawy T. E. (2014). Correlation between insulin, leptin and polycystic ovary syndrome. *Journal of Basic and Clinical Reproductive Sciences*.

[12] McGee W. K., Bishop C. V., Pohl C. R., Chang R. J., Marshall J. C., Pau F. K., Stouffer R. L., Cameron J. L. (2014). Effects of hyperandrogenemia and increased adiposity on reproductive and metabolic parameters in young adult female monkeys. *American Journal of Physiology: Endocrinology and Metabolism*.

[13] Arikan Ş., Bahceci M., Tuzcu A., Kale E., Gökalp D. (2010). Serum resistin and adiponectin levels in young non-obese women with polycystic ovary syndrome. *Gynecological Endocrinology*.

[14] Weker H., Laskowska-Klita T., Ambroszkiewicz J., Klemarczyk W., Marcinkowska M. (2001). Serum leptin level in prepubertal children with simple obesity. Part one. *Medycyna Wieku Rozwojowego*.

[15] Ramanand S. J., Ramanand J. B., Jain S. S. (2014). Leptin in non PCOS and PCOS women: a comparative study. *International Journal of Basic and Clinical Pharmacology*.

[16] Bideci A., Çamurdan M. O., Yeşilkaya E., Demirel F., Cinaz P. (2008). Serum ghrelin, leptin and resistin levels in adolescent girls with polycystic ovary syndrome. *Journal of Obstetrics and Gynaecology Research*.

[17] Olszanecka-Glinianowicz M., Madej P., Nylec M., Owczarek A., Szanecki W., Skałba P., Chudek J. (2013). Circulating apelin level in relation to nutritional status in polycystic ovary syndrome and its association with metabolic and hormonal disturbances. *Clinical Endocrinology*.

[18] Al-Watify D. G. O. (2014). Abnormalities of hormones and inflammatory cytokines in women affected with polycystic ovary syndrome. *Journal of Natural Sciences Research*.

[19] Tariq S., Tariq S., Alam S. S., Baig M. (2014). Effect of ibandronate therapy on serum homocysteine and leptin in postmenopausal osteoporotic females. *Osteoporosis International*.

[20] Kazmi A., Sattar A., Hashim R., Khan S. P., Younus M., Khan F. A. (2013). Serum leptin values in the healthy obese and non-obese subjects of Rawalpindi. *Journal of the Pakistan Medical Association*.

[21] Watanobe H. (2002). Leptin directly acts within the hypothalamus to stimulate gonadotropin-releasing hormone secretion *in vivo* in rats. *The Journal of Physiology*.

[22] Quennell J. H., Mulligan A. C., Tups A., Liu X., Phipps S. J., Kemp C. J., Herbison A. E., Grattan D. R., Anderson G. M. (2009). Leptin indirectly regulates gonadotropin-releasing hormone neuronal function. *Endocrinology*.

[23] Rehman R., Hussain Z., Faraz N. (2012). Effect of estradiol levels on pregnancy outcome in obese women. *Journal of Ayub Medical College, Abbottabad: JAMC*.

[24] Jockenhövel F., Blum W. F., Vogel E., Englaro P., Müller-Wieland D., Reinwein D., Rascher W., Krone W. (1997). Testosterone substitution normalizes elevated serum leptin levels in hypogonadal men. *Journal of Clinical Endocrinology and Metabolism*.

[25] Söderberg S., Olsson T., Eliasson M., Johnson O., Brismar K., Carlström K., Ahrén B. (2001). A strong association between biologically active testosterone and leptin in non-obese men and women is lost with increasing (central) adiposity. *International Journal of Obesity*.

[26] Sesmilo G., Casamitjana R., Halperin I., Gomis R., Vilardell E. (1998). Role of thyroid hormones on serum leptin levels. *European Journal of Endocrinology*.

[27] Oge A., Bayraktar F., Saygili F., Guney E., Demir S. (2005). TSH influences serum leptin levels independent of thyroid hormones in hypothyroid and hyperthyroid patients. *Endocrine Journal*.

[28] Koch C., Augustine R. A., Steger J., Ganjam G. K., Benzler J., Pracht C., Lowe C., Schwartz M. W., Shepherd P. R., Anderson G. M., Grattan D. R., Tups A. (2010). Leptin rapidly improves glucose homeostasis in obese mice by increasing hypothalamic insulin sensitivity. *The Journal of Neuroscience*.

